# Placing public health onto the Alzheimer’s disease and related dementias public policy platform

**DOI:** 10.1093/geront/gnaf224

**Published:** 2025-10-07

**Authors:** Brian Kaskie, Julie Bobitt, Yogesh Shah, Sarah Khasawinah

**Affiliations:** Department of Health Management and Policy, University of Iowa, Iowa City, Iowa, United States; Department of Medicine, University of Illinois Chicago, Chicago, Illinois, United States; Broadlawns Memory Center, Des Moines, Iowa, United States; Washington, DC, United States

**Keywords:** Alzheimer’s disease, BOLD Act, Dementia, Policy, Public health

## Abstract

In 2017, the United States Senate Special Committee on Aging added public health to the Alzheimer’s disease and related dementias (ADRD) policy platform by introducing the *Building Our Largest Dementia Infrastructure for Alzheimer’s Act*. Since then, 34 state health departments, 7 local, 2 territorial, and 1 tribal health organization have received a BOLD Program award from the Centers for Disease Control. With the support of the Alzheimer’s Association and university-based Centers of Excellence, their efforts have increased public awareness, expanded training of health-care providers, linked public health programs and health-care systems, and supported programs to reduce the risk for ADRD. In this forum, we draw on examples of federal and state policymaking targeting persons living with dementia and demonstrate how iron triangles consisting of advocacy organizations, public servants and policymakers have been critical in building a public policy platform for more than 50 years. We then consider how public health leadership may rely on such iron triangles to expand their role, focusing on the critical role assumed by professional and academic organizations in educating and training those who may help respond to the public health crisis being presented by the booming number of older Americans with ADRD.

## Placing public health onto the ADRD public policy platform

**Figure 1.**
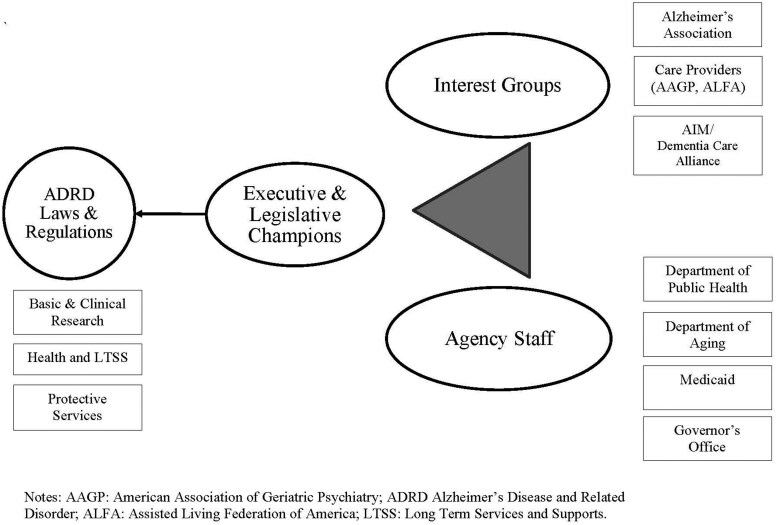
Iron triangle of ADRD policymaking.

In 2017, the United States Senate Special Committee on Aging (SSCA, 2017) added public health to the Alzheimer’s disease and related dementias (ADRD) policy platform by introducing the *Building Our Largest Dementia Infrastructure for Alzheimer’s Act* (United States Congress, P.L. 115-406). Upon passage in 2018, the Centers for Disease Control and Prevention (CDC) was authorized to provide funding to public health departments to establish and expand ADRD programs across state, local, territorial, and tribal jurisdictions (CDC, 2025). For the past 5 years, information about the prevalence of ADRD in local communities has been compiled, gaps in program and service delivery have been identified, public education has expanded, collaborations among administrative agencies have improved, and the provision of preventative care for persons living with dementia has grown (CDC, 2025). In December 2024, Congress reauthorized the BOLD Act (P.L. 118-142), extending the law through 2029 and requested $33 million to continue these remarkable efforts. As successful as the BOLD Act has been with placing public health onto the ADRD policy platform, such novel and valuable contributions require further investment. Public health leadership should consider the success associated with forming an “iron triangle” of policymaking to further develop, implement, and sustain public policies for persons living with dementia (Gais et al., 1983; Walker, 1969). Within such a policymaking triangle (see Figure 1), advocacy organizations mobilize public support and present priorities, legislative and executive staff provide technical knowledge and delineate regulation, and elected officials champion legislation and programs. Such collaboration ensures policies, such as those addressing ADRD, are supported and sustained over time (Volden & Shipan, 2017). Indeed, by forging such iron triangles among advocates, public servants, and elected officials, ADRD has remained a public policymaking priority for more than 50 years.

### Building the ADRD public policy platform

Fifty years ago, in 1974, Dr Robert Katzman conducted neurological autopsies of senescent older adults, repeatedly finding extensive degeneration and high concentrations of the hallmark plaques and tangles associated with Alzheimer’s disease. He recommended that senility no longer be defined as a normative age-related process (Katzman, 1976). During that same year, the United States Congress authorized the creation of the National Institute on Aging (NIA), and in 1978, the first national scientific symposium on Alzheimer’s disease was convened. The expert panel of research scientists and clinicians confirmed Alzheimer’s constituted an insidious pathology and they identified multiple etiological pathways (e.g., Lewy bodies, vascular infarctions) leading to age-related cognitive decline and other symptoms of dementia (Butler, 1984). NIA Director Robert Butler declared ADRD a significant public health problem. At the time, he forecasted ADRD would become one of the most devastating challenges of the 21st century as baby boomers advanced into older ages with an increasing probability of acquiring dementia relative to continuing decreases in mortality attributed to cancer, heart disease, and other conditions. By defining age-related declines in thinking and memory as a disease-driven pathological process and not a normal part of growing older, the need for public policymakers to support research and provide specialized care to persons living with dementia was established (Fox, 1989).

In 1980, Senator Thomas Eagleton (D-Missouri) chaired the first Congressional hearing on Alzheimer’s disease and called on leading researchers, clinicians, and caregivers of persons living with dementia brought forth by the Alzheimer’s Association (aka, Alzheimer’s Disease and Related Disorders Association, Inc.) to offer testimony about their lived experiences. The Committee recognized ADRD as a national public policy problem (Butler, 1984), and Congressional hearings have been convened almost every year since by the House Select Committee on Aging, the SSCA, and other standing committees. These public forums have brought forward advocates, researchers, and clinicians to describe the variety of challenges ADRD presents, and such testimony often has been followed by legislative staff and administrative agents developing viable public policy responses supported by elected officials who champion issues on behalf of persons living with dementia introducing bi-partisan legislation and regulations. The success associated with forming such iron triangles of ADRD policymaking has been exemplary.

In 1983, based on testimony provided by leading neurologists and psychiatrists, Congress moved to separate the diagnostic classification of persons living with dementia from those with primary psychiatric diagnoses, removing limitations on the provision of inpatient care (United States Congress, House Select Committee, 1992). The Health Services Research Extension Act of 1985 (United States Congress, P.L. 99-158, 1985) included funding to expand the national network of Alzheimer’s Disease Research Centers charged with finding a treatment or cure for ADRD as well as improving identification, diagnosis, and care. One of the first targeted pieces of ADRD legislation, the Alzheimer’s Disease and Related Dementias Research Act of 1986 (P.L., 99-160, 1986), called for a research advisory council within the Department of Health and Human Services that offered an annual progress report to Congress (Advisory Panel on Alzheimer’s Disease, 1992, 1995; National Institute on Aging, 2013). In 1987, Congress re-authorized the Older Americans Act and stipulated that the Administration on Aging conduct in-home supportive service demonstrations for individuals with dementia (Office of Technology & Assessment, 1990). Congress then moved to support state-wide service demonstrations focused on individuals with dementia and their informal caregivers (McConnell & Ruscio, 1991), and amendments included in the Omnibus Budget Reconciliation Act of 1989 expanded the network of qualified Medicare service providers as a means to increase access to diagnostic assessments and behavior management for persons living with dementia (Bartels & Colenda, 1998). By the end of the 1980s, the ADRD public policy platform was established (see Table 1).

**Table 1.** The ADRD public policy platform.

Date	Policy
**1966**	Medicare and Medicaid reimburses health-care and long-term services and supports provided to persons living with dementia.
**1972**	American Psychiatric Association does not recognize senile dementia as a non-normative aspect of advancing age.
**1974**	National Institute on Aging established; Alzheimer’s and other dementias *defined as neurologically-based diseases* characterized by multiple etiologies and insidious progression of thinking and memory loss.
**1978**	National Institute on Aging convenes research and clinical experts in first national symposium on Alzheimer’s disease and related dementias.
**1979**	Alzheimer’s Disease and Related Disorders Association aka Alzheimer’s Association incorporated.
**1980s**	*Every state established public education, information and referral services pertaining to Alzheimer’s disease*; more than half of the states form task forces on ADRD.
**1981**	First Congressional hearing on Alzheimer’s disease.
**1983–1989**	Key *federal legislation* supporting ADRD research and care, including:
	*1983-Separation of ADRD from psychiatric diagnoses in Medicare*
	*1986-Alzheimer’s Disease and Related Dementias Research Act*
	*1987-Omnibus Budget Reconciliation Act expands Medicare services*
**1990s**	Federal declaration of the “Decade of the Brain”; supporting research advancements in genetic and diagnostic research for ADRD.
**1990s**	*Every state established programs and services* targeting persons living with dementia and their care providers, through Medicaid, Older Americans Act, and state appropriations.
**2004**	*Alzheimer’s Disease Neuroimaging Initiative* launched; public-private research partnership administered by National Institute on Aging.
**2005**	CDC launches the *Healthy Brain Initiative*; begins the HBI Road Map Series linking public health with ADRD.
**2011**	*National Alzheimer’s Project Act (NAPA)* signed into law, establishing a coordinated national plan and increased funding for research.
**2016**	NAPA reauthorized, expanding efforts to offer evidence-based programs and services including informal caregivers.
**2017**	Senate Special Committee on Aging hearing focuses on ADRD risk reduction, diagnosis and system-level care improvements.
**2018**	*Building Our Largest Dementia Infrastructure* for Alzheimer’s Act (BOLD Act) signed into law.
**2019**	CDC aligns BOLD implementation efforts with the HBI Road Maps.
**2020–2024**	CDC awards funding to 43 public health departments.
**2024**	*BOLD Reauthorization Act* passed, extending the law through 2029

*Note.* ADRD = Alzheimer’s disease and related disorders; BOLD = Building Our Largest Dementia Infrastructure; CDC = Centers for Disease Control; HBI = Healthy Brain Initiative; NAPA = National Alzheimer’s Project Act.

### Expanding the ADRD public policy platform

As United States President George H.W. Bush (R) declared the 1990s as the “Decade of the Brain.” Congress directed the National Institutes of Health and other federal agencies to support research to identify risk factors, deterrents, disease-modifying therapies, and cures for neurological diseases and disorders, including ADRD (Proclamation 6158; July 19, 1990). As the decade concluded, researchers were developing reliable methods to identify those at higher risk for ADRD through genetic testing, analysis of abnormal brain activity, and the appearance of pre-dementia behavioral and cognitive symptoms (Hendrie et al., 2006). With such promising discoveries, the Alzheimer’s Association initiated an advocacy campaign to eliminate Alzheimer’s (“A World without Alzheimer’s”). The federal Alzheimer’s Disease Advisory Panel continued to offer annual reports to Congress highlighting promising discoveries and defining the most critical challenges in finding a cure or disease-modifying therapy for ADRD (Alzheimer’s Association, 2008; National Institute of Health, 2013; National Institute of Neurological Disorder and Stroke, 2014).

In 2004, the National Institute on Aging (NIA) launched the Alzheimer’s Disease Neuroimaging Initiative (ADNI, 2013; Potter et al., 2024). ADNI consisted of an unprecedented public-private biomedical research partnership proposed to find reliable methods to detect ADRD, distinguishing among varied biologically driven pathways preceding mild cognitive impairment, and track disease progression through moderate and severe stages. ADNI was originally funded at $60 million in 2004, with $40 million contributed from the NIA and $20 million from the pharmaceutical industry. By 2010, ADNI received an additional $24 million from the American Recovery and Reinvestment Act and an additional $70 million from its private supporters (ADNI, 2013).

In 2011, under the leadership of Chairman Herb Kohl (D-Wisconsin), the Senate Special Committee on Aging identified ADRD research funding as a bi-partisan priority, and Senators Evan Bayh (D-Indiana) and Susan Collins (R-Maine) advanced the National Alzheimer’s Project Act (NAPA; P.L. 111–375, 2011). NAPA sought to advance research across all federal agencies and accelerate efforts to identify cures or treatments to prevent, halt, or reverse the course of Alzheimer’s. In 2012, the National Institutes of Health (NIH) allocated an additional $50 million for Alzheimer’s disease research and then increased annual support by an additional $80 million in 2013.

The Reauthorization Act of 2024 extended NAPA through 2034 (P.L.118-92, 2024) increased annual research support, and expanded efforts to identify risk factors associated with cognitive decline, improve the quality and efficiency of care, increase education, and support protection of persons living with dementia through legal and fiduciary training programs (United States Department of Health and Human Services, 2024). The United States Congress authorized an annual allocation of approximately $3.7 billion for research dedicated to drug discovery, symptom management, caregiver support, and other critical issues relevant to persons living with dementia (National Institutes of Health, 2025). The NIA, in particular, currently supports 495 active clinical trials on ADRD; 68 of these are pharmacological trials, 157 non-pharmacological trials, 217 dementia care and caregiving trials, and 53 trials addressing other objectives, including public health strategies (https://www.nia.nih.gov/research/ongoing-AD-trials). Beyond research funding, the Department of Health and Human Services also finances programs and services provided to persons living with dementia through Medicare, Medicaid, and other federal, state, local, territorial and tribal agencies. The total annual cost of providing health-care and long term services and supports (LTSS) to persons living with dementia is estimated at $360 billion, and the Medicare and Medicaid insurance programs account for 64.0% of these payments (Alzheimer’s Disease Facts and Figures, 2024).

## Creating space for public health on the ADRD public policy platform

As the federal public policy platform expanded, an increasing number of researchers began to address public health issues concerning ADRD, focusing on potentially modifiable risk factors that may deter or delay the onset or progression of ADRD and other age-related chronic conditions such as heart disease, hypertension, diabetes, and high cholesterol (i.e., “what is good for the heart is good for the brain”). A growing body of evidence has suggested that as much as 45% of dementia may be attributable to potentially modifiable risk factors, including air pollution, depression, diabetes, excessive alcohol consumption, hearing impairment, hypertension, low social contact, obesity, physical inactivity, smoking, and traumatic brain injury (Livingston et al., 2024; World Health Organization, 2012). ADRD researchers also have created effective public education materials and training approaches for clinical care providers; affirmed evidence-based practices that identify and refer persons living with dementia to prevention programs, clinical care providers and support programs; and developed several non-pharmaceutical interventions that may reduce problematic symptoms and disease progression (Fried et al., 2023). Such research affirmed that essential public health services should be added to the ADRD policy platform.

Recognizing the critical role public health may assume for persons living with dementia, the CDC initiated a strategic effort to translate ADRD research discoveries into public health practice and convened a steering committee of national experts to lead the *Healthy Brain Initiative* in 2005 (Alzheimer’s Association & CDC, 2018). The initiative began by supporting state-wide surveillance projects (i.e., Behavioral Risk Factor Surveillance System) to define the prevalence of ADRD in state and local communities and identify needs for evidence-informed programs and services. Beyond conducting surveillance and sharing information about the prevalence and incidence of ADRD, the CDC published a series of Healthy Brain Initiative (HBI) Road Maps in 2007, 2013, 2018, and 2023 that presented actionable strategies for state, local and territorial public health departments to address cognitive impairment (CDC, 2013, 2025). Two additional HBI Road Maps have been published in 2019 and 2024 that present public health strategies to address cognitive impairment among American Indian and Alaska Native communities.

The HBI Road Map Series identified how efforts to address ADRD and improve the lives of persons living with dementia align with essential public health services (CDC, 2025). In particular, the CDC defined a place for public health departments to stand on the ADRD policy platform by: (a) supporting the development of evidence-based health promotion and prevention programs led by public health departments, (b) highlighting how public health departments may close gaps in care experienced by Persons living with dementia and their caregivers, and (c) demonstrating how community public health workers could offer public education, provider training, risk identification, referral services and prevention programs for persons living with dementia (DeSalvo et al., 2019; Olivari et al., 2020).

### The BOLD Act: putting public health on the ADRD policy platform

In March 2017, the Senate Special Committee on Aging (SSCA) convened “The Arc of Alzheimer’s: From Preventing Cognitive Decline in Americans to Assuring Quality Care for those Living with the Disease.” During the hearing, one expert witness discussed how modifiable risk factors could delay and deter the onset of ADRD, and another described how efforts by a large health system to educate clinicians to identify and refer persons living with dementia corresponded with improvements in care and reduced costs. Soon after, Chair Susan M. Collins (R-Maine) introduced the BOLD Infrastructure for Alzheimer’s Act with bipartisan support, and for the remainder of the 115th Congressional session, committee staff worked to educate members of Congress about the critical role assumed by state, local, territorial, and tribal public health departments. The BOLD Act was signed into law on December 31, 2018, with unanimous Senate approval and a 361-3 majority in the House of Representatives (United States Congress, P.L. 115-406).

In 2019, Congress allocated $10 million to the CDC to support state, local, territorial, and tribal efforts to compile and share information about ADRD; contribute to programs that reduce risk, delay onset and progression of ADRD; and public health departments were looked upon to convene meetings among state and local policymakers and other interested individuals and organizations. To support these implementation efforts, the CDC established three BOLD Public Health Centers of Excellence in 2020 with the first round of BOLD funding, and by 2023 as annual Congressional authorizations increased, the CDC had awarded cooperative agreements to 44 separate public health departments and one tribal health organization. The BOLD Infrastructure for Alzheimer’s Reauthorization Act (P.L.118-142, 2024) was passed unanimously in 2024 and extends the law through 2029. The Reauthorization Act renews support for several initiatives, including collecting data to track the incidence and prevalence of ADRD at state and local levels and increased collaborations with other state and local administrative agencies to promote brain health education, increase early detection of ADRD, and facilitate caregiver support.

### The iron triangle and ADRD policymaking

The passage and reauthorization of the BOLD Act affirms how the federal ADRD policy platform continues to be sustained and expanded by an iron triangle of policymaking consisting of advocacy organizations, legislative and executive staff, and elected officials (Gais et al., 1983; Walker, 1969). In this case, the 2017 SSCA hearing relied on witnesses to provide testimony about the challenges with identifying someone at-risk for ADRD. Since 1980, the Alzheimer’s Association and other advocacy organizations including the American Association of Geriatric Psychiatry and the Assisted Living Federation of America have sponsored witnesses who present policy agendas in committee hearings, task force gatherings, and office visits with elected officials (Kaskie et al., 2001). In 2010, such efforts were expanded further by the Alzheimer’s Impact Movement (AIM), a separately incorporated advocacy affiliate of the Alzheimer’s Association that educates members of Congress and other policymakers about the challenges presented by ADRD.

The 2017 SSCA hearing also relied on scientific experts who testified about the critical role that public health may play in the lives of persons living with dementia (SSCA, 2017). Such experts from the Department of Health and Human Services and other federal agencies have presented their latest research findings to members of Congress and identified viable policy alternatives to address the challenges presented by ADRD for more than four decades (Advisory Panel on Alzheimer’s Disease, 1995).

Last, this hearing highlighted the critical role assumed by senior policymakers such as Senators Collins (R-Maine) and Casey (D-Pennsylvania), who have championed issues on behalf of persons living with dementia for their entire careers and repeatedly have succeeded in enacting bipartisan legislation. By creating an iron triangle consisting of a range of advocacy interests, relying on leading scientific experts, and collaborating with administrative staff, the most senior members of Congress have built a robust, expansive, bipartisan ADRD public policy platform that now includes public health. Such a deliberate and inclusive approach to policymaking is exemplary and has been shown to result in continued reauthorizations and expanding support (Volden & Shipan, 2017).

## Expansion of the ADRD policy platform across the United States

ADRD public policymaking platforms have also been established within all 50 states (Kaskie et al., 2002; Office of Technology Assessment, 1990). For example, in addition to upholding more than 200 regulatory standards pertaining to the Nursing Home Reform Act of 1987, at least 30 states have expanded federal regulations to allow for the licensure and oversight of special care services offered to nursing facility residents with ADRD (Office of Technology Assessment, 1992). At least 10 states have enacted legislation supporting the establishment of adult day service programs and required secure perimeters to keep persons living with dementia from wandering (Kaskie et al., 2014). Almost every state has applied a Medicaid waiver designed to provide care for older adults with special needs, including persons living with dementia, who prefer to remain living at home in the community (MedPAC, 2013). The breadth of state ADRD policy platforms is extensive. State policymakers from both Democratic and Republican parties have addressed a range of regulatory challenges from supporting respite care programs to sponsoring law enforcement training about protective services for persons living with dementia (Kaskie et al., 2002).

These state ADRD policymaking efforts, like those at the federal level, have been sustained by iron triangles. The Alzheimer’s Association chapter network trains and supports volunteer advocates who meet with public officials about state and local challenges presented by ADRD (Alzheimer’s Association, 2008). Other organizations such as those representing nursing and assisted living facilities as well as home and community-based programs offering LTSS, also identify challenges presented by persons living with dementia and routinely engage with policymakers from both executive and legislative branches of state government (AIM, 2024). Like policymaking at the federal level, state level ADRD task forces have been critical for informing state policymaking (California Health and Human Services Agency, 2011; Office of Technology Assessment, 1990). State ADRD policy has been advanced by the most senior decision-makers, from both the Democratic and Republican parties, who forge bipartisan support to enact and sustain public policies to address challenges faced by persons living with dementia persons living with dementia, and many of these legislative and executive champions identify strongly with issues presented by ADRD because they have a family member living with ADRD (Kaskie et al., 2002).

### A place for public health departments on state ADRD policy platforms

As featured in this special issue of *The Gerontologist*, the BOLD Act has succeeded in placing public health departments across the United States onto the ADRD policy platform. Since 2020, 34 state health departments, 7 local health departments, 2 territorial health departments, and 1 tribal health organization have received a BOLD Program award from the CDC and agreed to engage in a multi-year process that begins by developing a strategic plan for implementing BOLD Program objectives (CDC, 2025). With the support of the CDC and three national Public Health Centers of Excellence, these public health efforts have increased awareness and understanding among the general public, expanded training of health-care providers and other professionals about ADRD, developed community-clinical linkages among public health programs and health-care systems, and supported programs proposing to reduce the risk for ADRD, promote early detection, and support dementia caregiving as guided by the HBI Road Map Series.

These localized BOLD Programs certainly are remarkable for their novel and varied approaches. Still, the need to increase and expand such efforts remains. No state, local, territorial or tribal public health department stands far above any other in terms of providing an exhaustive and exemplary set of dementia-specific risk reduction, early detection, or caregiver support programs or interventions. No single type of BOLD Program has been enacted nationwide, and no single department has implemented every type of program.

## Next steps for public health leadership

Current estimates suggest that 11.3% of Americans over age 65 experience ADRD, and one in every three persons over age 85 experiences some form of dementia. With the continued growth of America’s aging population, every state is expected to experience an increase of at least 5% in the number of persons living with dementia in the next decade (Dhana et al., 2023). Relative to this increasing growth, it has been established that providing essential public health services reduces the incidence of ADRD. The provision of essential public health services also has been shown to alter the clinical course of those who already experience ADRD and increasing the provision of such care has corresponded with improved outcomes and decreased expenditures (Beijani, 2023; Black, 2019; Bynum et al., 2014; Shehu et al., 2024). So, while securing a BOLD Program constitutes a notable advancement for public health departments and the BOLD Reauthorization Act sustains such efforts to raise awareness and improve care, the need to augment state, local, territorial, and tribal public health efforts relative to the pressures attributable to persons living with dementia remains.

One of the most distinguishing features of the HBI Road Map Series is the expectation that public health leaders align officials from governmental agencies which provide separately financed efforts to serve persons living with dementia including from aging services, Medicaid, and protective services (CDC, 2025). Once aligned, public health leaders are encouraged to then convene a network of formal care providers, including physicians, nurses, and home care aides; persons living with dementia and their caregivers; subject matter experts; and health-care and LTSS system leaders (i.e., bureaucratic experts, advocates). In some respects, the HBI Road Map Series leads to forging an iron triangle that maintains the place of public health departments on state, local, territorial, and tribal ADRD policy platforms.

### Establishing the value of public health

Now, with the BOLD Reauthorization Act extending the program another 5 years, public health departments have the opportunity to sustain and expand these efforts, learning from each other about how to secure funding and implement desirable programs. Leadership also may work to elevate public health efforts with state legislatures and seek to align with other established state efforts targeting older adults. In Iowa and Wisconsin, for example, legislators authorized funding to support the recruitment of dementia care specialists who work to advance efforts pertaining to risk reduction, service coordination, and caregiver support (State of Iowa, 2025; State of Wisconsin, 2025), and implementation of these efforts rely on the state wide network of Aging and Disability Resource Centers. The State of Illinois passed legislation in 2019 to establish a special state fund—the Alzheimer’s Disease Research, Care, and Support Fund—that Illinois taxpayers support through state income taxes which provides support for a full-time, state-level dementia coordinator, data collection on ADRD, and implementation of the Illinois Alzheimer’s Disease State Plan (State of Illinois 410 ILCS 410/3.2). Besides establishing a dedicated income tax check-off program, some states use civil monetary penalties collected from health-care and LTSS violations to support public health efforts to address ADRD (Miles, 2022).

Another way (see Table 2) for public health leadership to expand BOLD efforts is to align with existing prevention and promotion programs addressing age-related chronic conditions (e.g., cancer, heart disease, obesity) as supported by an appropriation from the Older Americans Act (Anderson et al., 2012). Several states have recently secured awards to establish Age-Friendly Public Health Systems (DeBiasi et al., 2020). The objectives of the overarching Age-Friendly Ecosystem movement align with the BOLD Act priorities as both seek to identify opportunities to coordinate efforts and implement sustainable, community-driven strategies for addressing ADRD.

**Table 2.** Next steps for public health leadership.

Goal	Action
**Public Health Departments**	
**Secure financial support**	BOLD program State allocation Tax CMP redistribution Foundation awards
**Align with existing efforts**	AOA/CDC funded prevention programs Foundation partnerships Age-friendly public health systems
**Collaborate across departments**	Medicaid Waiver support Multi-sector plan on aging
**Private and non-profit health system partnerships**	
**Medicare support services**	Staff dementia care specialists
**Medicare advantage**	Provide essential public health services Identification, referral, support
**Advocacy partnerships**	Information sharing Public education Provider training Agenda setting

*Note*. AOA = Administration on Aging; BOLD = Building Our Largest Dementia Infrastructure; CDC = Centers for Disease Control; CMP = civil monetary penalty.

BOLD Programs can be successfully expanded through collaborations with state Medicaid programs as well. One example of integrating public health with state Medicaid is CDC’s 6/18 Initiative, which connects health-care purchasers, payers, and providers with CDC researchers, economists, and policy analysts to find ways to improve health and control costs (CDC, 2025). In establishing the Age-Friendly Public Health Systems movement in 2018, Trust for America’s Health supported public health departments to develop and offer health and LTSS providers information about local populations of older adults, including persons living with dementia, who live at home alone in the community. Public health departments also can lead or contribute to community needs assessments, host public education events, support community-based primary and secondary prevention programs that address risk factors for and detection of ADRD, and disseminate information and referrals to tertiary preventions to delay disease progression or provide support to caregivers in managing the most challenging symptoms. More specifically, public health leadership may contribute to implementing Medicaid waivers designed to decrease residential placement and reduce the costs of care. By increasing education and training, expanding identification and referral, and supporting secondary and tertiary interventions targeting Medicaid beneficiaries with ADRD, such public health efforts may correspond with as much as a 33% reduction in spending for non-emergent emergency department visits and other avoidable health care and LTSS service spending attributable to persons living with dementia (Plichta, 2018).

One last way BOLD Program leadership can align with other government efforts targeting persons living with dementia is to participate in the state Multisector Plan for Aging (MPA) or another task force addressing older adults. Given how such efforts are often initiated by a governor’s executive order or legislative direction, the MPA framework authorizes the development of effective, customized strategies that align with state-specific needs and priorities (Rauscher et al., 2024). By including public health services with other agency efforts such as those developed by local law enforcement and emergency medical services, leadership can remain firmly positioned to offer unique and important contributions in addressing the challenges presented by persons living with dementia.

### Health system partnerships

In 2011, Medicare began reimbursing qualified providers to conduct an annual wellness examination that includes a basic evaluation to identify persons experiencing cognitive impairment (CMS, 2024). While most older persons prefer to have their memory and thinking evaluated by a qualified medical professional, less than one of every five have completed such basic evaluation (Jacobsen et al., 2019). In 2017, the Medicare program began reimbursing physicians, physician assistants, nurse practitioners, and clinical nurse specialists to develop a comprehensive dementia care plan (CMS, 2025). Such care planning can determine the need for dementia-specific services and refer persons living with dementia to community-based programs that can mitigate problematic symptoms, delay unwanted residential placement, and improve overall quality of life. Over the past 5 years, efforts to identify and refer persons living with dementia have grown, and the Alzheimer’s Association has developed a toolkit that helps provide direction to care providers.

Still, the need for health systems to increase public education, provider training, identification, early detection, and care planning remains. Such efforts may be increased by aligning health and LTSS systems with state, local, territorial, and tribal public health departments offering information about local rates of ADRD, providing public and patient education, provider training and resources, and facilitating access to caregiver support as well as referrals to secondary and tertiary services. As we look to the future, the reauthorization of the BOLD Act reinforces the vital role of public health in addressing ADRD. For the past 10 years, state, local, territorial and tribal public health departments have developed and disseminated essential tools such as surveillance systems, messaging strategies, and community-based models, that now position other sectors to step into support efforts targeting persons living with dementia and their care providers.

In some respects, just as public health played a foundational role in raising awareness about cancer prevention and the importance of early screening, it was ultimately the health-care system, particularly primary care, that operationalized these strategies through delivery of services. A similar handoff is needed in the context of dementia: healthcare professionals, integrated systems, and community health providers must now adopt and embed these public health strategies into routine care. The continued success of these efforts will depend on the strength of established iron triangles bringing together advocates, executive agencies, and legislative champions to sustain and expand dementia-focused public health initiatives. By leveraging this proven policymaking framework, public health leaders can support a broader ecosystem of actors working toward a shared vision of healthier aging.

Medicare-managed care plans, in particular, may be more likely to support such efforts to conduct risk identification, needs assessments, and care planning for persons living with dementia because they are reimbursed and financially rewarded for doing so (United Health Group, 2007). Medicare Advantage plans can reimburse for case management, adult day care, respite for caregivers, in-home meal delivery, and other LTSS valued by persons living with dementia and their caregivers. The provision of these services reduces the inefficient use of emergency care, ambulatory sensitive hospital admissions, and low-acuity nursing facility placements—decreasing aggregate health-care spending and increasing Medicare providers’ profit margins (Shehu et al., 2024). Alternatively, upon identifying someone at-risk for ADRD and receiving an increased payment for doing so, Medicare-managed care plans may also avoid providing additional services to persons living with dementia as they are considered clinically more complex, often presenting with multiple conditions that require continued personal care. Even so, access to local, evidence-based programs and services for all Medicare beneficiaries including those enrolled in managed care remains limited (Thomas et al., 2019). This, too, may be of interest to public health leaders.

### Advocacy alliances

Besides continuing to work with the Alzheimer’s Association and university partners with implementation of the BOLD Act, public health leaders also can look to engage with health policy issue networks that engage and inform senior legislative champions. In 2020, the Milken Institute Center for the Future of Aging (Super et al., 2022) launched the Alliance to Improve Dementia Care (the “Alliance”). The Alliance positioned itself at the intersection of health, business, and policy, and recruited several advocacy groups (e.g., the AARP, Alzheimer’s Association, LEAD Coalition, UsAgainstAlzheimer’s) to champion legislative and regulatory initiatives to improve the lives of persons living with dementia and their caregivers. The Alliance includes more than 90 organizations and pharmaceutical companies. Most relevant here, the Alliance declared interest in collaborating with the CDC BOLD Public Health Centers of Excellence and engaging policymakers through policy briefs, op-eds, and virtual briefings. However, it remains unclear how many public health leaders have engaged with the Alliance. An initial first step might be for public health leaders to engage with the Alliance to identify members of state, local, territorial, and tribal governments who champion issues pertaining to ADRD and would be interested in learning more about the BOLD Program and forging an iron triangle of ADRD policymaking.

## Concluding remarks

Given the absence of a federally approved cure or disease-modifying therapy, the persistence of ADRD and the associated costs of care are devolving into a national calamity. Recent efforts to place public health on the ADRD policy platform have been remarkable but there is still much work to do. Perhaps this call to public health leadership to prioritize efforts focusing on persons living with dementia may be more favorably received if amplified by leadership of organizations that educate and train public health professionals. The American Public Health Association identifies the aging population as a topic of interest and recently produced a report focusing on the relationship between social determinants of health and successful aging and specifically called for improvements in the dementia care workforce. Such efforts to focus public health professionals certainly warrant continued support relative to the booming population of aging Americans, particularly those over 85 years old, who will suffer with ADRD in the decade ahead (APHA, 2024). America’s growing population of persons living with dementia and their informal care partners should become a more visible priority among those who are responsible for educating and training the next generation of public health professionals. At this time, among more than 170 American colleges and universities conferring degrees in public health, less than 10% offer a degree or training in aging, gerontology, cognitive health or a field related to ADRD (Kaskie & LePore, 2023). This lack of education and training seems conspicuous given how the population of persons living with dementia is expected to reach 10.0 m by 2035. In fact, as we look to the future, efforts to prepare the next generation of public health leaders who can sustain the momentum generated by the Reauthorization of the BOLD Act most certainly has reached a critical point.

## Data Availability

This article does not report data and therefore the data availability requirements are not applicable. This study is not registered. **Supplement sponsorship** This article appears as part of the supplement “Healthy Brain Initiative (HBI) and Building Our Largest Dementia Infrastructure (BOLD): Dementia as a Public Health Imperative,” supported by the Centers for Disease Control and Prevention of the U.S. Department of Health and Human Services (HHS) as part of a financial assistance award totaling $14,229,665 with 100% funded by CDC/HHS. The contents are those of the author(s) and do not necessarily represent the official views of, nor an endorsement, by CDC/HHS, or the U.S. Government.
